# Conifer R2R3-MYB transcription factors: sequence analyses and gene expression in wood-forming tissues of white spruce (*Picea glauca*)

**DOI:** 10.1186/1471-2229-7-17

**Published:** 2007-03-30

**Authors:** Frank Bedon, Jacqueline Grima-Pettenati, John Mackay

**Affiliations:** 1 Centre d'étude de la Forêt, Université Laval, Pavillon Charles-Eugène Marchand, Sainte Foy G1K7P4, Québec, Canada; 2 UMR CNRS/UPS 5546 Surfaces Cellulaires et Signalisation chez les Végétaux, Pôle de Biotechnologie Végétale, BP426 17 – Auzeville 31226, Castanet Tolosan, France

## Abstract

**Background:**

Several members of the R2R3-MYB family of transcription factors act as regulators of lignin and phenylpropanoid metabolism during wood formation in angiosperm and gymnosperm plants. The angiosperm *Arabidopsis *has over one hundred *R2R3-MYBs *genes; however, only a few members of this family have been discovered in gymnosperms.

**Results:**

We isolated and characterised full-length cDNAs encoding *R2R3-MYB *genes from the gymnosperms white spruce, *Picea glauca *(13 sequences), and loblolly pine, *Pinus taeda *L. (five sequences). Sequence similarities and phylogenetic analyses placed the spruce and pine sequences in diverse subgroups of the large R2R3-MYB family, although several of the sequences clustered closely together. We searched the highly variable C-terminal region of diverse plant MYBs for conserved amino acid sequences and identified 20 motifs in the spruce MYBs, nine of which have not previously been reported and three of which are specific to conifers. The number and length of the introns in spruce *MYB *genes varied significantly, but their positions were well conserved relative to angiosperm *MYB *genes. Quantitative RTPCR of *MYB *genes transcript abundance in root and stem tissues revealed diverse expression patterns; three *MYB *genes were preferentially expressed in secondary xylem, whereas others were preferentially expressed in phloem or were ubiquitous. The *MYB *genes expressed in xylem, and three others, were up-regulated in the compression wood of leaning trees within 76 hours of induction.

**Conclusion:**

Our survey of 18 conifer *R2R3-MYB *genes clearly showed a gene family structure similar to that of Arabidopsis. Three of the sequences are likely to play a role in lignin metabolism and/or wood formation in gymnosperm trees, including a close homolog of the loblolly pine *PtMYB4*, shown to regulate lignin biosynthesis in transgenic tobacco.

## Background

Insights into the regulation of lignin biosynthesis during vascular development of plants are being derived from angiosperm model plants like *Arabidopsis thaliana *(reviewed by [1]) and from investigations unravelling the molecular basis of wood formation in trees like *Populus *(reviewed by [2]). Members of the R2R3-MYB transcription factor family have been implicated as regulators of phenylpropanoid and lignin metabolism [1] as well as pattern formation and differentiation of primary and secondary vascular tissues, (reviewed by [3]). The MYB proteins comprise one of the largest families of plant transcription factors, which is represented by over one hundred members in the model plant *Arabidopsis *[4]. The biological roles of MYBs have been deduced primarily from flowering plants (angiosperms) including snapdragon [5], maize [6], *Arabidopsis *[7,8] and eucalyptus [9]. By contrast, the biological roles of only a few R2R3-MYBs has been examined in non-flowering plants (gymnosperms) and relatively little is known about of their gene family structure [10-12]. The loblolly pine genes Pt*MYB1 *and Pt*MYB4 *were shown to be transcriptional activators which have the ability to regulate lignin synthesis enzymes [10,11]. They are expressed in xylem tissues, bind AC elements and activate transcription in transient assays in yeast or plant cells [10,11,13]. Overexpression of pine *MYB*s resulted in ectopic lignification in tobacco [10] and in *Arabidopsis *[8] The reports are strong evidence supporting a role for MYBs in the lignifying process in gymnosperm trees. Lignins play an important role in trees because they confer rigidity and impermeability to wood by accumulating in thickened secondary vascular tissues [1,14], therefore its regulation is of interest for understanding the genetic basis of wood properties. However, the number of *MYB *transcription factors that may participate in regulating lignification in gymnosperms, and their potential roles remain an open question.

Gymnosperms, especially conifers of the *Pinaceae *familyare ecologically and economically important due to their abundance in forests in many parts of the world (North America, Europe, Asia) and because of their use in diverse wood products (pulp and paper, solid wood and engineered lumber). Despite recent large-scale gene discovery initiatives for conifer trees like pine and spruce (e.g. [15,16]), only a few regulatory gene families have been characterised systematically in any conifer species. In one such study, it was recently shown that the structure of the *knox-I *gene family appears to be monophyletic in the *Pinacea*, whereas angiosperms have several distinct clades (four in dicots and three in monocots) [17]. The R2R3-MYB family is very large with over 120 members in angiosperms [4] and has been divided into several subgroups [18,19]. One may predict that several MYBs are likely to regulate lignin metabolism and other aspects of wood formation in conifer trees; however no data have been available from which to infer the size or the structure of the family in gymnosperms. Therefore, a broader survey of *MYB *genes expressed in the vascular and other tissues of gymnosperms seems essential for developing a better understanding of their roles in gymnosperm lignin biosynthesis and wood formation.

MYB proteins have two structural regions, an N-terminal DNA-binding domain (DBD or MYB domain) and a C-terminal modulator region that is responsible for the regulatory activity of the protein. The MYB domain is well conserved among plants, yeast and animals [20]. Its consensus sequence contains around 50 amino acid residues with regularly spaced tryptophans giving rise to a helix-turn-helix structure [21]. There are usually one to three imperfect repeats of the MYB domain. Proteins with two repeats (R2R3-MYBs) are specific to plants and yeast [22] and are the most abundant type in plants [4]. Plant R2R3-MYBs take part in many biological processes including seed development and germination [23], the stress response [24] and epidermal cell fate in addition to their involvement in phenylpropanoid and lignin biosynthesis [5,8-11] and vascular organisation [3] (for a review, see Ref. [25]).

The genetic selection and breeding activities of a few commercial conifer species are being expanded to include genetic mapping and marker development. Candidate gene approaches are being adopted to identify robust genetic markers derived from genes that have a physiological role in the traits that are targeted by breeders [26]. Our goal is to characterise several members of a gene family proposed to play a role in controlling lignin synthesis and wood properties in conifer trees, in order to support candidate gene approaches for marker discovery. In this report, we characterize 13 different *R2R3-MYB *gene sequences from the white spruce, *Picea glauca*, (designated *PgMYB*) and five from loblolly pine, *Pinus taeda L *(designated *PtMYB*). The full-length coding sequences we obtained enabled us to explore their phylogenetic relationships to other plant *MYB *genes and to search for novel amino acid motifs within this large protein family. We also compared the gene structures, i.e. number, size, position and splice sequences of introns, to gain further insights into their evolution. The steady-state levels of *MYB *and cell wall-related gene mRNAs were examined by Q-RTPCR in various spruce tissues and organs with an emphasis on wood-forming tissues and compression wood formation. We identify three *MYBs *that are preferentially expressed in secondary xylem and are also upregulated during the formation of compression wood.

## Results

### Isolation and sequence analysis of 18 *R2R3-MYB *genes from spruce and pine

**Figure 1:**
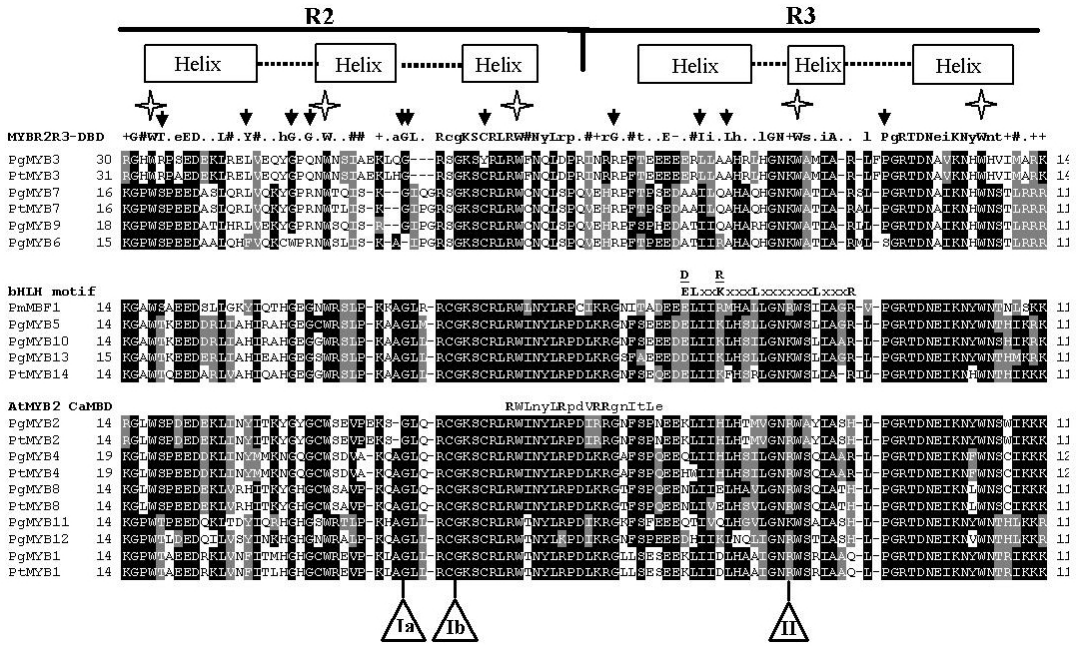
**Alignment of predicted MYB domain protein sequences from spruce and pine**. Amino acid sequence alignments of the 21 conifer MYB R2R3 domains were obtained with Clustal W (see Methods) and then separated into three groups based on their homologies to the consensus R2R3-MYB DNA-binding domain (MYBR2R3-DBD, top panel), the bHLH protein-binding motif (bHLH motif, middle panel) or the *Arabidopsis *calmodulin-interaction motif (AtMYB2 CaMBD, bottom panel), as indicated. Black shading indicates identical amino acid residues and grey shading the similar residues that agree with the fraction sequence of 0,4 (BoxShade 3.21) and dashes indicate gaps. The numbers on the left and right indicate the amino acid position relative to the translation start codon. The boxes and dotted line above the sequences show the predicted helix and turn structures in the R2 and R3 regions of the MYB domain. Stars show positions of conserved tryptophan residues and black arrows indicate unusual amino acid residues compared to the consensus amino acid sequence of the MYB DNA-binding domains of several plant R2R3-MYB proteins described by Avila *et al*. [27]. The bHLH protein-binding motif ([DE]L × 2 [RK] × 3L × 6L × 3R) identified by Zimmerman *et al*. [28] and the calmodulin-interaction motif [29] are shown above the middle and bottom panels, respectively (major amino acids in upper-case, bold). Ia or Ib and II indicate the positions of the first and second introns, respectively (Ib is specific to *PgMYB3*). Accession numbers of the newly identified spruce and pine MYBs are listed in Methods. Pg, *Picea glauca*; Pt, *Pinus taeda*; Pm, *Picea mariana*; At, *Arabidopsis thaliana*.

We isolated and sequenced 18 full-length cDNAs encoding *R2R3-MYB *genes from conifer trees: 13 from spruce (*PgMYB1-13*) and five from pine (*PtMYB2, 3, 7, 8 *and *14*). Each of the full length cDNA sequences were obtained starting from partial or full length clones identified by EST database mining (all of the pine sequences and most of the spruce sequences), or starting from the pine sequence and using RT-PCR amplification with conserved primers to amplify a spruce fragment (Table 1). For partial clones, we used RACE cloning to identify flanking sequences and, full length PCR amplification to generate a single full length cDNA. Their predicted amino acid sequences were aligned together with the three available full-length MYB sequences from gymnosperms [10-12]. The DNA-binding domains of these 21 gymnosperm sequences showed a high level of amino acid conservation particularly in the R3 helix-turn-helix repeat, consistent with its involvement in DNA binding (Fig. 1). Most of the variations among the spruce *PgMYB *sequences were located in the turn of each R repeat.

The conifer sequences were consistent with the consensus DBD sequence identified by Avila *et al*. [27], which was largely based on angiosperm sequences. Only a few amino acid residues differed from this consensus; these were mainly in *PgMYB 3, 6, 7 *and *9 *and *PtMYB3 *(black arrows in Fig. 1). We found a motif similar to that involved in the interaction with basic helix-loop-helix (bHLH) proteins in *Arabidopsis *([DE]L × 2 [RK] × 3L × 6L × 3R; [28]) in the R3 repeat of three spruce MYBs (*PgMYB5, 10 *and *13*) as well as in *PmMBF1 *([12]; Fig. 1). *PtMYB14 *had a similar motif but with two differences: an R instead of an L, and a gap before the last R residue. In addition, several conifer *MYBs*, including those with the bHLH motif (except *PmMBF1*), encoded an R × 5R × 3RR motif similar to the calmodulin-interaction site previously described in the DBD of *Arabidopsis MYB2 *[29]. The highest level of conservation with the calmodulin-binding motif was observed in *PgMYB2 *and *PtMYB2*, but most of the conifer *MYB *genes shown in Figure 1 had a similar motif.

### Phylogenetic relationships and gene family structure of conifer R2R3-MYBs

**Figure 2:**
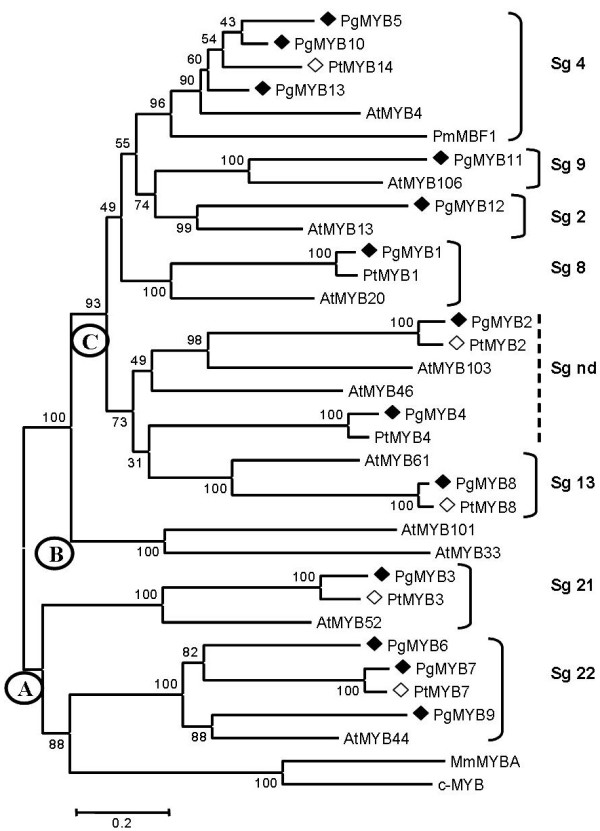
**Phylogenetic tree of gymnosperm and angiosperm R2R3-MYB proteins**. This neighbour-joining (1000 Bootstraps) tree was based on the Clustal W alignment of the complete coding sequences of 13 spruce and five pine MYB proteins identified in this study (represented by filled and empty lozenges, respectively). The bar indicates an evolutionary distance of 0.2%. *Arabidopsis *proteins were chosen as landmarks representing the three main groups (circles A, B and C) and subgroups (Sg next to bracket; nd, not determined) defined by Romero *et al*. [19] and Kranz *et al*. [18]. Human c-MYB [GenBank: P10242] and *Mus musculus *MmMYBA [GenBank: X82327] were not used as out groups but as landmarks. The accession numbers of the *Arabidopsis *genes are given in Methods. Other abbreviations are in Figure 1.

We used the Mega 2.0 method to construct a phylogenetic tree using full length cDNA sequences (Fig. 2). The result of our analysis is congruent with the three major groups of R2R3-MYBs (A, B and C) defined by Romero *et al*. [19] on the basis of their binding affinities to MYB recognition elements. On this phylogenetic tree, the predicted spruce MYB proteins sequences fell into several subgroups with bootstrap values ranging from 96–100%, indicating the high grouping robustness. All of the spruce and pine MYB sequences fell into group A (PgMYB3, 6, 7 and 9, and putative pine orthologues) or C (all the other conifer sequences in Fig. 2) and none belonged to the B group. The conifer sequences were assigned to 7 of the 22 subgroups previously defined based upon *Arabidopsis *sequences [18]. Four of the conifer sequences (PgMYB2 and 4; PtMYB2 and 4) clustered with *Arabidopsis *sequences that do not fit into a defined subgroup. Several pairs of spruce and pine sequences clustered closely together with short branch-lengths indicative of a high degree of homology (Fig. 2). Indeed, pair-wise optimal alignments with the Clustal W algorithm of the pine and spruce pairs 1, 2, 3, 4, 7 and 8 gave amino acid identities from 95% to 100% for the DBD and of 79%–93% for the complete coding sequence (Table 3), suggesting that they are putative orthologous pairs. By comparison, PtMYB14 was less homologous to its neighbouring spruce sequences PgMYB5, 10 and 13 (60% to 67% homologous for the full CDS).

We also analysed the number, size and sequences of introns in PCR-amplified genomic DNA, as a complement to the phylogenetic analysis based on the coding sequences. In angiosperm R2R3-MYBs the introns are located in the Myb DBD, therefore we sequenced this specific region in genomic DNAs of the 13 spruce *R2R3-MYBs*, isolated by PCR amplification with gene specific primer pairs spanning each gene's coding region (Additional file 1). Most of the gDNA sequences were identical to the cDNAs, ranging from 100% to 99.3% in amino acid identities (data not shown), due to a few variations in the predicted amino acid sequences. The sequences were also verified for the lack of non-sense mutations (stop codons or frameshifts). As observed in angiosperms, we found spruce *MYB *genes with one (I), two (I, II) or no introns (Table 2).

Similarity was found between the spruce MYBs in terms of intron position, phases and, in some cases, between intron sequences, but the number and length intron was quite variable. Generally, the second intron (II) was longer than the first (I) except in *PgMYB11 *where intron I was five times longer than intron II. The spruce sequences belonging to group A MYBs fell into two subfamilies with distinct gene structures, i.e. with one intron (Sg21) and one without introns (Sg22). The group C sequences all had one or two introns, as in *Arabidopsis *[30]. The intron I occurred before the GL amino acid pair in repeat 2 and the intron II occurred after the GN amino acid pair in repeat 3 (Fig. 1), as found in the majority of *Arabidopsis R2R3-MYB *genes[30]. Only *PgMYB3 *had a different intron I site, named Ib, located before the GKS amino acids. Moreover, the phase (1 or 2) of insertion was consistent, and the end sequences (GT in 5' and AG in 3') were conserved among the sequences we analysed (Table 2). Phylogenetically close sequences, like *PgMYB5*, *10 *and *13*, had similar 5' and 3' splice junctions for both introns (Table 2). The intron of these three genes also showed strong nucleotide sequence conservation, although the first intron of *PgMYB5 *was much longer due to a 20-nucleotide triplicated sequence (not shown).

### Sequence analysis of conserved regions in the C-terminal of *P. glauca *MYBs

The coding regions of the spruce *PgMYB *sequences ranged widely in length, encoding between 196–526 amino acid residues depending on the length of the C-terminal region (Table 1). We used the predicted C-terminal coding regions of the spruce MYB proteins to search for conserved sequences, reasoning that such motifs might be important for the function or post-translational regulation of MYB. We used the MEME motif-detection software to analyse the C-terminal region of spruce MYBs using a set of protein sequences selected for their high degree of similarity to each of the spruce MYBs (Table 1, Additional files 2 and 3). Our approach incorporated a large diversity of sequences; it identified a total of 20 different motifs (A-T) in the spruce MYBs, including nine new unpublished motifs (Table 1) and 11 that were reported previously [18,19,30]. The probability scores for each of the motifs identified in this study ranged from 2.39^e-09 ^to 1.79^e-18^. The lowest previously published score for such motif was 6.79^e-12 ^(motif S in PgMYB11) [30]. We detected between zero (in PgMYB4) and five (in PgMYB9) motifs per protein in the predicated spruce MYB sequences. The large number of conifer sequences enabled us to detect three amino acids regions, I, K and P, that appeared to be specific to gymnosperms (in PgMYB6, 7 and 9). Other motifs, such as F and G, were shared between gymnosperm and angiosperm sequences. Four of the conserved amino acid sequences (A, F, L and Q) shared the central core residues GIDPxTH but displayed differences in neighbouring amino acids between the consensus sequences of subgroups 4, 8, 9 and 13 defined by Kranz *et al*. [18].

### Expression of *P. glauca *MYB genes in tissues of young and mature trees

**Figure 3:**
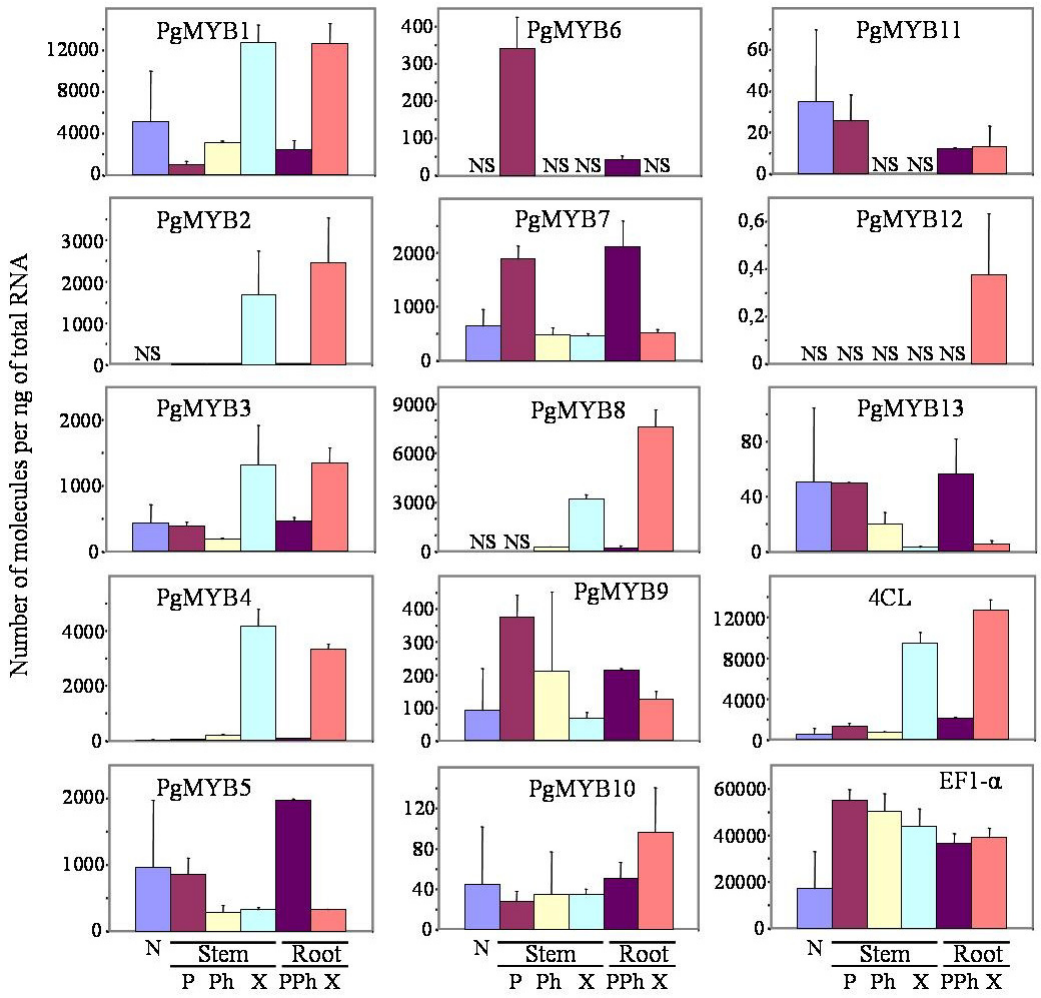
**Transcript abundance for 13 spruce *MYB *genes and *4CL *in various organs and tissues**. Transcript abundance was determined by Q-RTPCR of six tissues from two different 33-year-old trees (number of molecules per ng of total RNA, see methods). The transcript level of an *elongation factor *(*EF1-α*) gene was used as an RNA control. N, needles; Stem tissues: P, periderm; Ph, differentiating phloem; X, differentiating xylem; Root tissues: PPh, root periderm with differentiating phloem; X, root differentiating xylem. Data are based on three technical repetitions per tree, i.e. six measurements per data point. Vertical bars represent the standard error. *4CL*: *4-coumarate: CoA ligase*. NS, no PCR product detected.

We surveyed the abundance of each of the 13 *PgMYB *gene transcripts by Q-RTPCR, in mature (33-year-old trees) and young (3-year-old) green-house-grown trees to determine their tissue distribution during normal development. Six different organs and differentiating tissues (the young needles; the periderm, phloem and xylem from the stems; and the periderm with phloem, or bark, and xylem from the roots) were collected from two different mature trees (Fig. 3). For tissue comparisons, we calculated the number initial *MYB *RNA molecules per ng of total RNA. Spruce *PgMYBs 2*, *4 *and *8 *were expressed preferentially in differentiating xylem from stem and root. Other *MYBs *were abundant in the needles along with one to two other tissues from the stem or the roots or both, Some *MYB *mRNAs also appeared to have rather ubiquitous profiles or low abundance transcripts. The RNA abundance of lignin biosynthesis enzymes PAL, 4CL, CCoAOMT and CAD were also determined in the same tissue samples. The lignin enzymes RNAs all gave very similar profiles, and they were most abundant in differentiating xylem (only *4CL *is shown; Fig. 3).

**Figure 4:**
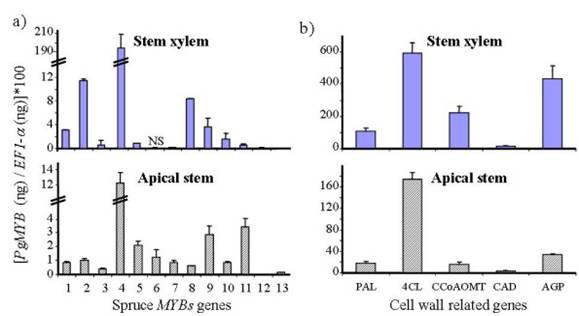
**Transcript abundance for *MYB *genes and secondary cell-wall-related genes in differentiating secondary xylem and in primary growth (new flush) of spruce seedlings**. Transcript abundance was determined as in Figure 4 for, a) 13 spruce *MYB *genes, and b) five cell-wall-related genes in differentiating secondary xylem from stem and in the elongating terminal leader (apical stem) from 3-year-old spruce seedlings. The standard error (bars) was calculated from three biological replicates and two independent technical repetitions (i.e. six independent measurements). *PAL*, *phenylalanine ammonia lyase*; *4CL*, *4-coumarate*: *CoA ligase*; *CCOaOMT*, *caffeoyl-CoA 3-O-methyltransferase*; *AGP*, *arabinogalactan protein; CAD*, *cinnamyl alcohol dehydrogenase*. NS, no PCR product detected.

We also compared the abundance of the different *MYB *transcripts in the differentiating secondary xylem and in the elongating apical leader of young spruce trees (Fig. 4). The cell wall-related genes *PAL*, *4CL*, *CAD*, *CCoAOMT *and an *arabinogalactan protein *(*AGP*) were included in this analysis. For these within tissue comparisons, the data were normalized against the *EF1-α *transcript levels. Again, the spruce *MYB *transcripts *2*, *4 *and *8 *were clearly the most abundant among the MYBs detected in the secondary xylem, consistent with the data from the mature trees. In the apical leader, the relative abundance of the *MYB *transcripts was quite different than in the secondary xylem, except that *PgMYB4 *transcripts remained very abundant. Some *MYB *genes that were weakly expressed or not detectable in secondary xylem were among the most highly expressed in apical stem (*PgMYB6*, *7 *and *11*; Fig. 4a).

### Spruce *MYB *genes are differentially expressed in compression wood

**Figure 5:**
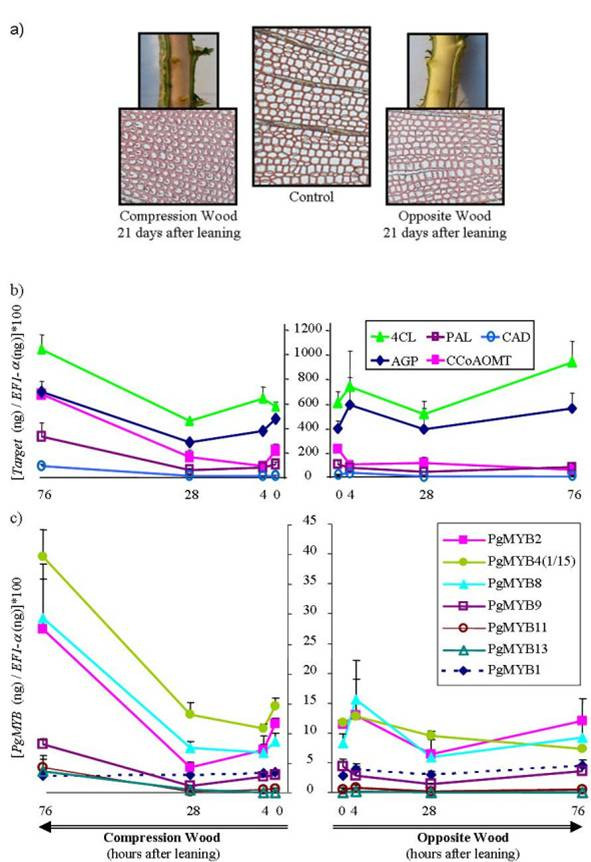
**Transcript accumulation for *MYB *genes and secondary cell-wall-related genes in differentiating compression wood and opposite wood**. a) Compression wood and opposite wood formed in a leaning spruce seedling after 21 days of treatment, compared to the control from vertical seedling. Exposed wood (compression wood is light brown) and wood cross-sections (10 μm thick) were stained by the safranin-orange procedure [53] (magnification, ×40). Steady-state mRNA levels were determined as in Figures 4 and 5 for cell-wall-related genes (b) and for several *PgMYB *genes (c) in the compression wood (left panels) and opposite side wood (right panels) of spruce seedlings leaning at a 45° angle from vertical. Continuous lines indicate genes with significant variation, and standard error bars are shown three trees (biological replicates) with two independent technical repetitions). Discontinuous lines indicate examples of gene transcripts that do not fluctuate in abundance. The zero time point represents vertical control trees only. *PgMYB4 *(1/15) means that mRNA level is divided by 15.

We followed the expression of the 13 spruce *MYB *genes and five cell-wall-related genes during the early phases of compression wood formation, in order to explore further the potential involvement of MYBs in wood formation and lignin biosynthesis. Gymnosperm trees form a type of reaction wood (known as compression wood) on the lower side of a bent or leaning stem, or in branches. Compression wood is enriched in lignin and contains lignins that are more condensed. Therefore compression wood formation requires the modulation of lignin biosynthesis, which we hypothesized to involve such gene sequences as R2R3-*MYBs*. We induced the formation of compression wood in actively growing 3-year-old spruces by maintaining at a 45° angle (relative to vertical) (Fig. 5). After 21 days of growth in this leaning position, characteristic compression wood was well developed on the lower side of the stems (Fig. 5a). We chose to monitor transcript abundance over a 76-hour period immediately after induction, and found that several transcripts accumulated between 28 and 76 hours (Fig. 5c). The transcripts of *PgMYB2*, *4 *and *8 *clearly increased in the xylem forming compression wood compared to the opposite wood and compared to the vertical trees (0 hour time point). The transcripts of *PgMYB9*, *11 *and *13 *RNA were slightly increased and the seven others did not fluctuate significantly. By contrast, no significant variation in spruce *MYB *RNA abundance was observed in the opposite wood, which is found on the upper side of the stem (opposite to the the compression wood). Transcripts for *PAL*, *4CL*, *CCoAOMT *and *CAD *lignin biosynthesis enzymes as well as the *AGP *also increased within the same time-frame as the *MYB *transcripts (Fig. 5b). In the opposite wood, only *CCoAOMT *RNA transcripts decreased. No significant variation in transcript abundance was observed for the spruce *MYB *or lignin genes in the terminal shoots of the same seedlings (data not shown).

## Discussion

In this paper, we report the complete coding sequences of 18 conifer gene sequences that share the characteristic features of the *R2R3-MYB *gene family. Thirteen sequences were from *P. glauca *(white spruce; *PgMYBs*) and five from *P. taeda *L. (loblolly pine; *PtMYBs*). We characterised the full-length cDNA sequences, as well as the spruce exon-intron structure. We assigned the conifer sequences to several phylogenetic clades of the R2R3-MYB family and identified conserved motifs within them based on predicted amino acid sequences. The steady-state mRNA levels of spruce MYBs were surveyed in several tissues to identify those genes that are preferentially expressed in wood-forming tissues. Furthermore, we identified *PgMYBs *whose transcript levels are upregulated, along with those of an *AGP *and enzymes of lignin biosynthesis, during the induction of compression wood in young spruce trees.

### Sequence conservation and identification of amino acid motifs in spruce R2R3-MYBs

Our data show that the DBDs of conifer MYBs are highly conserved, whereas the C-terminal region are highly variable, as shown in prior studies of other plant MYBs. The predicted amino acid sequences of some of the spruce MYB DBDs contain a motif for interaction with bHLH proteins and/or with calmodulin. We identified twenty amino acid motifs in the variable C-terminal region, of which nine were previously unreported. The amino acid motifs in the DBD and in the C-terminal region are useful to better characterise the spruce R2R3-MYB sequences belonging to each phylogenetic clade.

The R2R3-MYBs are specific to plants and are subdivided into three major groups according to their binding affinities [19]. The more than 120 *Arabidopsis *sequences were placed into 22 subgroups based on their overall amino acid sequences and C-terminal motifs [18]. Amino acid motifs are conserved among members of several of the 22 phylogenetic clades or subgroups [18,30,31], including the bHLH-interaction and calmodulin-binding motifs in the DNA-binding domain [28,29], and the repression domain pdLNLD/ELxiG/S in the C-terminus [7]. In our study, the spruce group C sequences were dispersed among seven phylogenetics clades, five of which were previously defined as distinct subgroups by Kranz *et al*[18]. All the spruce members of subgroup 4 harboured the bHLH-interaction motif as well as the C-terminal motifs F and G, except for PmMBF1 [12], which lacked the motif G (pdLNLD/ELxiG/S) described by Kranz *et al*[18]. The bHLH-interaction motif identified by Zimmermann *et al*. [28] is required for MYB proteins to transactivate some of the phenylpropanoid and anthocyanin genes through protein-protein interactions [32]. The G motif in the C-terminus has been linked to transcriptional repression of the *cinnamate 4-hydroxylase *(*C4H*) gene by AtMYB4 [7]. Several genes in group C also encoded a conserved GIDP sequence located after the end of the DBD, suggesting a conserved molecular function for this motif. The DBDs of PgMYB2, 4 and 8, which were upregulated during compression wood formation, harboured a motif similar to the calmodulin-interaction site of AtMYB2 [29], suggesting a potential link with the calcium signalling pathway implicated in the regulation of secondary wall formation [33]. No conserved regions were detected in the C-terminal region of PgMYB4 and its closest homolog PtMYB4, even though experimental evidences indicate that PtMYB4 is a regulator of lignin synthesis enzymes [10], as is the case for the closely related EgMYB2 [9]. The presence of a regulator motif in PgMYB4 may have escaped our analysis because the parameters were set to detect motifs ranging from 5–15 amino acids in length; motifs of less than five amino acids or scattered in several small modules may thus remain undetected.

Spruce MYBs were relatively under-represented in group A, where they fell into subgroups 21 and 22. In our analysis, spruce group A MYBs contained six of the nine newly identified C-terminal consensus amino acid sequences. Three of these motifs were specific to conifers assigned to subgroup 22: motifs I, K and P found in PgMYB6, 7 and 9. The motifs might be involved in protein or DNA interactions; however, it remains to be seen whether they play a role in protein structure or function.

### Spruce MYB phylogeny and evolution

There are very few reports from which to estimate the number of *R2R3-MYB *genes in gymnosperms or to gain insights into the molecular evolution of this protein family [10-12,34,35]. According to the phylogenetic relationship with other *MYB *genes in angiosperms and gymnosperms, the spruce MYB sequences described here belong to nine different MYB clades distributed between group A and group C described by Romero *et al*. [19]. None of the conifer sequences identified in this study and none of the reported gymnosperm R2R3-MYBs were assigned to the B group [19]. We may hypothesize that group B sequences are present only in angiosperms, however, more gene discovery work is needed to draw conclusions since only four of the 125 *Arabidopsis MYB *genes belong to this group B [19,31].

Despite recent large-scale gene discovery initiatives for conifers like pine and spruce (e.g. [15,16]), only a few regulatory gene families have been characterised in any conifer species. The *R2R3-MYBs *family has evolved and expanded very rapidly through numerous gene duplications in Angiosperms [36]. Given the very distant separation of gymnosperms and angiosperms (approx. 300 million years), we were interested in assessing whether a similar gene family evolution would be present in both taxonomic groups. In other words, is the *R2R3-MYB *gene family structure similar in these two groups? In the *knox-I *gene family of conifer trees, the structure and number of genes was shown to be very different from than of angiosperms, in a recent study investigating evolution of the family in great detail [17]. Several of the angiosperm clades are missing in conifers which appear to have undergone several recent gene duplications with relatively low sequence divergence levels. Our work provides a clear indication that the conifer MYB family structure is not all that divergent from that of the angiosperms, in contrast to the Knox-I report, suggesting that the basic family structure predates the gymnosperm – angiosperm split. In maize, several subgroups of *R2R3-MYB *genes have expanded within the past 50 million years [36,37]. Consistent with this, our analysis of coding sequences and introns in spruce *MYB *genes also suggests more recent gene duplications in, at least in some of the clades. For example, *PgMYB5*, *10 *and *13 *have high levels of nucleotide sequence similarity in coding sequence as well as introns I and II.

Further investigation is needed to discover the full complement of conifer *MYB *sequences. By comparison to the angiosperms, we predict that the set of sequences described here represents a fraction of the conifer R2R3-MYB family. Identification of new sequences would complete the evolutionary picture of this conspicuous family of regulators and help to determine its position in the evolution of plant lineages.

### Potential involvement of the spruce *R2R3-MYBs *in the lignification of woody tissue

The spruce and pine sequences we analysed represent diverse subgroups of the R2R3-MYB family. Thus, we hypothesized that they could play diverse roles in metabolism and development. The involvement of specific *R2R3-MYB *gene products in lignin biosynthesis and/or wood formation is suggested by their expression profiles and by their sequence homology with genes from pine ([10,11]) and in other species whose functions have been previously tested. The AC *cis*-regulatory elements, for example, which are found in many promoters of phenylpropanoid and lignin biosynthesis genes, play an important role in gene regulation in lignifying xylem cells, thus linking *R2R3-MYB *genes with lignin biosynthesis. AC elements have been implicated in the transcriptional regulation of *PAL *in bean [38], *4CL *in parsley [39], *CCR *and *CAD *in *Eucalyptus *[40,41].

We compared the abundance of the 13 different spruce *MYB *mRNAs in selected tissues and organs that develop a secondary vasculature in mature spruce, and in the primary stems and differentiating secondary xylem in young trees. *PgMYB2*, *4 *and *8*, all of which belong to the same phylogenetic clade, were expressed preferentially in the secondary differentiating xylem of both juvenile plants and mature trees. Interestingly, all three genes were also expressed preferentially in xylem tissues isolated from large roots. By comparison, *4CL *had a very similar transcript profile in prospected tissues. The other *MYB *genes had various patterns of expression including phloem-preferential and ubiquitous patterns.

We also compared the RNA levels in differentiating secondary xylem during the induction of compression wood in spruce seedlings. Compression wood development in conifers that are leaning or bent is characterised by the formation of thicker cell walls, increased lignin content and the deposition of more condensed lignin polymers, among other features [42]. The plasticity of lignin biosynthesis and cell wall architecture observed in compression wood have been linked to the fluctuation in abundance of several gene transcripts and proteins [43,44]. Although the transcriptional regulators that orchestrate this plasticity are unknown, they might include MYB transcription factors due to their implication in xylem differentiation and in lignin biosynthesis. The three *PgMYBs *(*2*, *4 *and *8*) that were preferentially expressed in xylem are likely candidates because they were also upregulated on the compression wood-forming side (downward side) of the stem but remained relatively constant on the opposite side. The time-course and relative magnitude of the changes in transcript levels of the *PgMYBs 2*, *4 *and *8 *were quite similar to those seen for genes encoding lignin biosynthesis enzymes and an *AGP *surveyed in the same samples. Three other *PgMYBs *(*9*, *11 *and *13*) also showed an increase in transcript abundance in secondary xylem upon induction of compression wood. In the control trees, the mRNA for *PgMYB11 *was one of the highest we examined in the apical portion of the stem but it was low in secondary xylem. These observations might imply a role for *PgMYB11 *in processes that are common to primary stem growth and compression wood formation. The *MIXTA *gene from *Antirrhinum majus*, which belongs to the same phylogenetic subgroup, is involved in cellular development [45] and may provide clues to the role of *PgMYB11 *in conifers.

The expression profiles of a few of the spruce *MYB *genes are consistent with previous reports describing the putative function of homologous genes. For example, spruce *PgMYB4 *is a close homolog of *PtMYB4 *[10], which induced ectopic lignification when overexpressed in transgenic tobacco. A putative role for *PgMYB4 *in lignification is consistent with our data showing a higher mRNA level in compression wood, characterised by increased lignin deposition. The gene *PgMYB8 *(subgroup 13) showed strong similarity with *AtMYB61*, which is expressed in xylem tissues of Arabidopsis and was shown to play an important role in regulating lignification [8]. *AtMYB61 *is also expressed in developing seeds, where it regulates the extrusion of seed coat-derived rhamnogalacturonan mucilage [23]. The accumulation of *PgMYB8 *transcripts in compression wood is consistent with the ectopic lignification resulting from the constitutive overexpression of *AtMYB61 *in *Arabidopsis *[8]. By contrast, *AtMYB103*, the most similar *Arabidopsis *sequence to *PgMYB2*, is not expressed in the stem but is involved in trichome and tapetum development [46], suggesting a putative role in xylem differentiation other than lignin biosynthesis.

The spruce sequence *PgMYB1 *has a close pine homolog, *PtMYB1*, that has been linked to lignin biosynthesis [11], however the spruce sequence was not expressed preferentially in secondary xylem (it was also expressed in needles, phloem and the shoot apex) nor was it induced during compression wood formation. It was demonstrated that *PtMYB1 *is able to bind the AC-I and AC-II elements (PAL-Box) [11]. Recently, Gomez-Maldonaldo *et al*. [13] showed that pine MYB1 and MYB4 bind to glutamine synthetase AC elements and that MYBs are linked to several metabolic pathways by shared *cis*-acting elements. Based on these observations, it appears that the *MYB1 *genes of pine and spruce may regulate phenylpropanoid metabolism as well as nitrogen assimilation in various plant tissues.

## Conclusion

Through a systematic survey of EST sequence data followed by full length sequencing, we characterised 18 conifer *R2R3-MYB *gene sequences (13 from *P. glauca*, white spruce; 5 from *P. taeda*, loblolly pine). Three *R2R3-MYBs *from spruce, namely *MYB 2*, *4 *and *8 *were shown to be expressed preferentially in secondary xylem. We also found that transcript levels of six *PgMYB *genes (including the *MYB 2*, *4 *and *8 *genes), were upregulated in differentiating secondary xylem from young trees during the induction of compression wood along with cell-wall-related genes. Our study highlights a small set of spruce *MYB *transcription factors that could be good candidate genes for marker development studies. Gain-of-function/loss-of-function studies using transgenic plants are also needed to delineate the roles of these different MYBs. Such studies are expected to lead to greatly lacking insights into the regulation of wood formation in conifers.

## Methods

### Plant material and RNA isolation

Several tissues were isolated from two 33-year-old *P. glauca *trees felled in July 2003, from a progeny trial established near Quebec City (Canada). All tissues were frozen in liquid nitrogen immediately upon removal from the tree and stored at -80°C until further use. We collected newly formed needles from the upper crown. Differentiating secondary xylem and phloem, as well as bark tissues were collected from three 30–40 cm bolts taken from the lower third of the main stem. These vascular tissues were scraped with a scalpel immediately after peeling the bark. Tissues scrapped from the exposed inner side of the bark and from surface of the exposed wood were labelled as differentiating secondary phloem and xylem, respectively. Similarly, differentiating xylem and bark (including phloem) were collected from large roots located in a one-meter radius from the base of the stem. Samples from each tree and each tissue were kept separate for RNA extraction and gene expression studies.

A gravitropic treatment to induce compression wood formation was performed on 3-year-old spruce seedling stock. The seedlings were transferred to 3 L pots one month before the experiment, grown in a greenhouse with 16 hours light per day, and fertilised weekly with 20 g/L N-P-K. A randomised design of 24 young trees was established in which 12 trees were maintained at 45° angle by leaning the pots and tying the plants to stakes (also at 45°); 12 seedlings were grown in the normal vertical position. Destructive tissue samplings were carried out 4, 28 and 76 hours after the beginning of the treatment. The average diameter of the plants near the base was 7.2 +/- 0.61 mm, their average height was 60.63 +/- 7.25 cm, and the terminal leader was 19.83 +/- 2.91 cm. For each time point, four vertical and four leaning trees were harvested mid-morning in a randomised order, and three randomly selected trees were used for gene expression analyses. Secondary xylem was collected as described above from the two sides of the main stem of the seedlings; the lower and upper sides representing compression wood and opposite side wood, respectively, for the leaning trees, or left and right side for vertical tree. The whole terminal leader was also collected from each plant. Total RNAs were isolated from tissues described above and ground in liquid nitrogen with a pestle and mortar, except for the gravitropic treatment where a Mixer Mill MM300 engine (Retsch) was used to grind in Microtubes (Eppendorf). The RNAs were extracted from each tissue sample and each tree or seedling separately, following the procedure of Chang *et al*. [47], in Oakridge tubes or in Microtubes. RNA concentration and quality were determined with a bioAnalyser (model 2100, Agilent Technologies; RNA 6000 Nano Assay kit).

### DNA cloning and accession numbers

Previous reports described two complete coding sequences of *R2R3-MYB *genes from pine (*PtMYB1 *and *PtMYB4*) [10,11] and one from spruce [12] as well as several partial sequences [35] expressed in xylem tissues of pine. We isolated partial spruce cDNA clones representing putative orthologues of the pine *MYB *genes by PCR amplification, and identified several partial and putative full-length sequences among the spruce EST sequences data of the ARBOREA project derived from 17 different cDNA libraries[48] For each of the partial spruce and pine gene sequences, we obtained complete coding sequence and UTRs by using 3' RACE, 5' RACE or both cloning methods on spruce or pine mRNA from needles or xylem (SMART RACE cDNA Amplification Kit, Invitrogen, Carlsbad, CA). DNA was cloned in pCR2.1 with the TA cloning Kit (Invitrogen, Carlsbad, CA) and sequenced. The sequence analyses presented hereafter are based upon cDNA clones containing the complete coding sequences of the *MYB*, which were isolated as a single fragment from reverse transcribed RNA, with gene specific primer pairs for each of the 13 sequences from spruce and five sequences from pine. The numbering of pine *MYB *genes (*PtMYB1-8 *and *PtMYB14*; no *PtMYB5 *and *6 *reported) is in accordance with Patzlaff *et al*., [10,11]; the numbering of spruce genes was established on the basis of putative orthology with the pine sequences.

In addition, we isolated the 13 corresponding sequences from spruce genomic DNA (gDNA). Genomic DNA was extracted from needles of white spruce using the Genomic-Tip Kit (Qiagen, Mississauga, Ontario). The entire coding region with introns was isolated by PCR amplification with gene specific primer pairs spanning each gene's coding region (Additional file 1) and cloned in pCR2.1 with the TA cloning Kit (Invitrogen, Carlsbad, CA). The gDNA was from *Picea glauca *genotype Pg653 and so did most of the cDNA clones (although a few came from wild *Picea glauca *genotypes). Each clone was sequenced at least through the MYB DBD in order to determine the number of introns present in this region. Some nucleotide differences were observed between cDNA and gDNA sequences due to the genotypic variation, but no non-sense mutation were detected. The genomic sequences of *PgMYB *3, 6, 7 and 11 showed 1 to 3 non synonymous substitutions giving no less than 99.2% amino acids identity; however, we do not find nucleotide mismatches in spruce *MYB *2, 4, 8, 9 and 12.

The 13 *MYB *genes from spruce and five *MYB *genes from pine have the following accession numbers: *PgMYB1 *[GenBank: DQ399073], *PgMYB2 *[GenBank: DQ399072], *PgMYB3 *[GenBank: DQ399071], *PgMYB4 *[GenBank: DQ399070], *PgMYB5 *[GenBank: DQ399069], *PgMYB6 *[GenBank: DQ399068], *PgMYB7 *[GenBank: DQ399067], *PgMYB8 *[GenBank: DQ399066], *PgMYB9 *[GenBank: DQ399065], *PgMYB10 *[GenBank: DQ399064], *PgMYB11 *[GenBank: DQ399063], *PgMYB12 *[GenBank: DQ399062], *PgMYB13 *[GenBank: DQ399061] and *PtMYB2 *[GenBank: DQ399060], *PtMYB3 *[GenBank: DQ399059], *PtMYB7 *[GenBank: DQ399058], *PtMYB8 *[GenBank: DQ399057], *PtMYB14 *[GenBank: DQ399056].

The nucleotides sequences of candidate genes involved in wood formation come from the spruce EST assembly directory number 8 (dir8) of the ARBOREA project[48] Their percentage amino acid sequence similarity with other species is given in brackets. They are:

*phenylalanine ammonia lyase *(*PAL*) [dir8: contig10199] partial coding sequence (cds), 85% to *Pinus taeda *[GenBank: U39792];

*4-coumarate: CoA ligase *(*4CL*) [dir8: contig10433] partial cds, 86% to *Pinus taeda *[GenBank: U39405];

*caffeoyl-CoA 3-O-methyltransferase *(*CCOaOMT*) [dir8: contig5884] complete cds, 92 % to *Pinus taeda *[GenBank: AF036095];

*arabinogalactan protein *(*AGP*) [dir8: contig10745] complete cds, 75 % to *Pinus taeda *[GenBank: U09556];

*cinnamyl alcohol dehydrogenase *(*CAD*) [dir8: contig9065] complete cds, 95 % to *Pinus radiata *[GenBank: AF060491],

and the housekeeping gene *elongation factor alpha *(*EF1-α*) [dir8: contig10829] complete cds, 99% to *Picea abies *[GenBank: AJ132534].

### Sequence analyses and phylogenetic studies

Nucleotide and amino acid sequence alignments were obtained with Clustal W [49] using BioEdit software version 6.0.7 and BoxShade 3.21 to highlight sequences. Delineation of introns was achieved by aligning the cDNA and genomic nucleotide sequences of the *PgMYB *from the start codon to stop codon on the basis that introns begin with GT and end with AG dinucleotides. Phylogenetic studies were performed with the 13 predicted MYB protein sequences from white spruce, one sequence from black spruce (PmMBF1, [12]), and seven loblolly pine sequences including previously reported PtMYB1 and 4 [10,11]. We also included 11 diverse *Arabidopsis *MYB sequences and two *R1R2R3-MYB *genes from human and mouse as landmarks to classify the MYBs according to previous reports [18,19,30]. We constructed a neighbour-joining tree based on a Clustal W amino acid alignment generated with the Mega 2.0 method [50] and using 1000 bootstraps to estimate the node strength (parameters are Poisson correction and pair-wise deletion as described in [30]).

The accession numbers of the *Arabidopsis *genes analysed are: AtMYB13 [GenBank: At1g06180], PtMYB1 [GenBank: AY356372], AtMYB4 [GenBank: At4g38620], AtMYB103 [GenBank: At1g63910], AtMYB46 [GenBank: At5g12870], PtMYB4 [GenBank: AY356371], AtMYB61 [GenBank: At1g09540], AtMYB52 [GenBank: At1g17950], AtMYB44 [GenBank: At5g67300], AtMYB20 [GenBank: At1g66230], AtMYB101 [GenBank: At2g32460], AtMYB106 [GenBank: At3g01140], AtMYB33 [GenBank: At5g06100], PmMBF1 [GenBank: U39448]. The accession numbers of the conifer MYB genes are as above.

MEME analysis software [51] was used to identify amino acid regions conserved between several members of a subgroup of sequences (according to Kranz *et al*. [18]) containing one or more spruce MYBs. The parameters setting was the number of motifs to find: 5; minimum width of motif: 5 and maximum: 15. We used complete protein sequences of MYBs from 12 conifer species and from 14 angiosperm species (Additional file 2). We also included 10 partial conifer sequences that encompassed the C-terminal region [34] and were closely related to PgMYB6, 7 and 9 (Additional file 3).

### Analysis of transcript accumulation by Q-RTPCR

To analyse the transcript abundance of *PgMYBs*, first-strand cDNA was synthesised starting from one microgram of RNA treated with amplification grade DNAse I (Invitrogen) and purified on an RNeasy column (Qiagen), using oligo(dT) primers and SuperScript II RT (Invitrogen) according to the manufacturer's instructions. The resulting cDNA was diluted 1:5 with sterile RNAse-free water and stored at -20°C; the same cDNA was used for all Q-RTPCR (quantitative reverse transcription PCR) assays for any given tissue sample. Steady-state mRNA levels were determined on cDNA by quantitative RT-PCR using Opticon Monitor 2 fluorescence detection system (MJ research) and DyNAmo SYBR Green QPCR kit (Finnzymes Oy, Espoo, Finland). Gene primer pairs were designed using the Primer 3 software [52] to anneal near the 3' end of each transcript (usually in 3' UTR) to ensure primer specificity. The forward and reverse primers were as follows (amplicon length indicated in brackets):

*PgMYB1*: 5'-gattgtacattaacccagtaa-3' and 5'-taaaccatgtggtatctgtta-3' (148 bp);

*PgMYB2*: 5'-tgggtattctaggtatttcc-3' and 5'-attaggtaagtatgcaggg-3' (99 bp);

*PgMYB3*: 5'-agatcacggacccagatcaac-3' and 5'-gagcgaacgacctccttcag-3' (147 bp);

*PgMYB4*: 5'-gcagtttgagtttgagtgtg-3' and 5'-ctggagcatagatttgatga-3' (162 bp);

*PgMYB5*: 5'-aattctggcagcgaactg-3' and 5'-aatgcttcgtggtggaatc-3' (173 bp);

*PgMYB6*: 5'-tttccttccttcatttcaac-3' and 5'-taaatttgggtttctgttgc-3' (108 bp);

*PgMYB7*: 5'-tcgagttgcacatcaggag-3' and 5'-gagtgtggatggcaaacag-3' (156 bp);

*PgMYB8*: 5'-ggtggactcagttgtaataa-3' and 5'-gtatctcacctatttacagatca-3' (101 bp);

*PgMYB9*: 5'-gaaattcgagaaacatggtg-3' and 5'-aaacgacagaaatcgagaac-3' (149 bp);

*PgMYB10*: 5'-gctgtattttaacatttcatgg-3' and 5'-acaacaatctttctttttctcc-3' (133 bp);

*PgMYB11*: 5'-cccagcttatgactggaag-3' and 5'-tacagaacaaccatgcagac-3' (157 bp);

*PgMYB12*: 5'-caggtgacttaactctattccag-3' and 5'-tcacatagaacaggcatgg-3' (143 bp);

*PgMYB13*: 5'-aaattacagctagagtgagagg-3' and 5'-aacttgaaccgtacacgac-3' (86 bp) and

*EF1-α *(elongation factor alpha): 5'-aactggagaaggaacccaag-3' and 5'-aacgacccaatggaggatac-3', (114 bp). Forward and reverse primer pairs used for the Q-RTPCR of the wood formation-related genes are:

*PAL*: 5'-tggatttgcatcctactg-3' and 5'-tccatcttcaactataggac-3' (103 bp);

*4CL*: 5'-cattcctcaaaagcatgaagag-3' and 5'-atcgcatccacaaagtacac-3' (150 bp);

*CCOaOMT*: 5'-attgagatcagccaaatcc-3' and 5'-gcgctctccctataatcag-3' (124 bp);

*AGP*: 5'-gcgtccattgttttaatgtag-3' and 5'-tgtatttatccctctgtctgc-3' (181 bp) and

*CAD*: 5'-ctggactacatcaatactgc-3' and 5'-gatttactcattctgcacg-3' (141 bp).

The PCR reaction mixture (20 μL) consisted of 10 μL DyNAmo SYBR Green qPCR mix, 1 μL primers (0.25 μM forward and 0.25 μM reverse), 1 μL of cDNA and 8 μL of RNase-free water. The cycling conditions were 95°C for 2 min, then 35 cycles of 95°C for 10 sec, 55°C for 10 sec, 72°C for 8 sec, followed by a plate reading. The melting curve readings were carried out every 0.2°C from 65 to 95°C, holding 1 sec at each temperature. Standard curves were established for each spruce *MYB *and cell-wall-related cDNAs and were used to determine RNA abundance in each sample. The standards consisted of serial dilutions of PCR amplicons prepared from each cDNA, cloned in pCR2.1, with M13 reverse and forward primers. Amplicon standards were gel purified (Qiagen), and product length and concentration were verified using a bioAnalyser (model 2100 Agilent Technologies, DNA 1000 LabChip kit). The standard curves were determined from duplicate reactions from the dilutions series of each amplicon. Raw data were converted with the following parameters: no blank subtraction, subtract baseline and average over cycle range according each case. We calculated the number of transcript molecules per ng of total RNA [(DNA quantity quantified in g * DNA base pair mass per gram of DNA)/M13 Reverse-Forward amplified fragment length in bp]. For within tissue comparisons that were carried on differentiation xylem (including compression wood induction) and apical shoots young trees, the RNA abundance was normalised using the abundance of RNA of the elongation factor alpha (*EF1-α*), as an endogenous control (calculated as the following ratio: *target gene *(ng)/*EF1-α *(ng)).

## Abbreviations

Q-RTPCR, quantitative reverse transcriptase polymerase chain reaction; EST, expressed sequence tag; RACE, rapid amplification of cDNA-ends.

## Authors' contributions

FB carried out the experimental work and the data analysis, participated in the design of the study and drafted the manuscript. JGP and JM participated in the design of the study and in manuscript preparation. All authors read and approved the final manuscript.

## Supplementary Material

Additional file 1 Primers sequences of spruce *MYBs *used for genomic amplification and sequencing. The table provides the nucleotides sequences of the primers with respective Tm values and amplified genomics DNA length, for each spruce *MYB *genes. Click here for file

Additional file 2 Phylogenetic tree of MYBs from spruce, pine and nearest sequences from other species. The figure shows the phylogenetic relationship between conifers MYBs and others MYBs on the basis of their complete amino acids sequences. Protein sequences from each clade incorporating spruce MYBs was used to search for conserved amino acid motifs. Click here for file

Additional file 3 Conifer MYB phylogeny based on partial sequences using a 31 amino acids region. The figure shows the phylogenetic relationship between several conifers MYBs on the basis of a 31 amino acid region in the third MYB domain repeat. Due to the availability of many partial sequences, this short region leads to the addition of several conifer sequences. Click here for file

## Figures and Tables

**Table 1:** Predicted lengths and C-terminal motifs of spruce MYB proteins

Sg^1^		Full length cDNA^2^	DNA Binding Domain^3^	C-terminal Domain^3^	Motifs	Consensus sequences	Angio-Gymno^4^	MEME E-value	Start motif^5^	Ref.
4	Pg MYB5	-b-	115	142	F	LlsrGiDP(at)tHrp(li)n	13/13-5/5	6.00^e-14^	1	a)
					G	e(re)cpdLNLel(cr)ispp	13/13-4/5	3.31^e-16^	67	a), b)
	Pg MYB10	-a-	115	95	F	LlsrGiDP(at)tHrp(li)n	13/13-5/5	6.52^e-14^	1	a)
					G	e(re)cpdLNLel(cr)ispp	13/13-4/5	4.32^e-15^	67	a), b)
	Pg MYB13	-b-	116	80	F	LlsrGiDP(at)tHrp(li)n	13/13-5/5	1.18^e-14^	1	a)
					G	e(re)cpdLNLel(cr)ispp	13/13-4/5	5.43^e-14^	65	a), b)
22	Pg MYB6	-a-	115	235	H	(cs)s(sv)DPpT(ls)LsLslPg	7/7-14/14	2.02^e-14^	99	d)
					I	YlkaedaismmsaAv	0/7-13/14	1.87^e-13^	141	d)
					J	vmremvakEVrsYmn	7/7-14/14	1.07^e-17^	188	a), b), c)
	Pg MYB7	-c-	116	257	K	egdyEVesrgLKRln	0/7-13/14	1.34^e-12^	43	d)
					H	(cs)s(sv)DPpT(ls)LsLslPg	7/7-14/14	4.28^e-13^	113	d)
					I	YlkaedaismmsaAv	0/7-13/14	2.30^e-10^	161	d)
					J	vmremvakEVrsYmn	7/7-14/14	6.15^e-16^	206	a), b), c)
	Pg MYB9	-a-	119	297	K	egdyEVesrgLKRln	0/7-13/14	6.03^e-16^	60	d)
					P	hRQSAFksYesqktp	0/7-11/14	1.19^e-13^	116	d)
					H	(cs)s(sv)DPpT(ls)LsLslPg	7/7-14/14	2.91^e-13^	144	d)
					I	YlkaedaismmsaAv	0/7-13/14	9.50^e-16^	205	d)
					J	vmremvakEVrsYmn	7/7-14/14	1.09^e-16^	256	a), b), c)
8	Pg MYB1	-b-	115	217	A	lr(kq)mGiDP(lv)THkpl	5/5-2/2	1.79^e-18^	1	a)
21	Pg MYB3	-c-	130	177	C	(fg)Re(rq)S(rs)(is)(rg)(kr)R	4/5-2/2	4.69^e-14^	1	d)
					D	e(en)s(l)(vs)(pt)ffDfl(g)vG(cn)	5/5-2/2	1.26^e-13^	35	a), b)
					E	(cy)xi(sg)h(in)nh(v)q(sf)(jr)Kef	3/5-2/2	4.76^e-14^	123	d)
13	Pg MYB8	-c-	115	411	L	LrrGIDP(n)THkpl	4/4-2/2	2.54^e-17^	1	a)
					M	VC(dv)(yk)(np)SIm(al)nPsm(yn)	2/4-2/2	1.94^e-18^	199	d)
					N	e(ye)(ae)vKWSEml	2/4-2/2	6.45^e-14^	317	d)
					O	(pk)D(fl)(hq)R(im)Aa(vs)(lf)(dg)q	2/4-2/2	4.89^e-15^	399	a)
9	Pg MYB11	-a-	115	384	Q	L(lv)kMGIDPvTHkp(k)	6/6-1/1	4.08^e-16^	1	a), b), c)
					R	h(m)AQWEsARleAear	6/6-1/1	3.10^e-13^	35	a), b), c)
					S	(yc)eDnknYw(nd)silnlV	4/6-1/1	6.79^e-12^	360	c)
2	Pg MYB12	-a-	115	254	T	MdfW(fl)(dn)v(fl)(t)	5/5-1/1	2.39^e-09^	237	a)
nd	Pg MYB2	-c-	115	333	B	(c)SylPPL(y)d(v)	2/2-2/2	3.29^e-13^	249	d)
	Pg MYB4	-c-	120	214	none	none	0/3-0/2	none	none	none

Conserved amino acid regions were identified in angiosperm and gymnosperm C-terminal sequences by the use of MEME software (setting described in Methods). Motifs were detected among the sequences belonging to each phylogenetic clade comprised of at least one spruce MYB (Additional File 2). Sequences from Additional File 3 were used to identify more conifer members of the PgMYB6, 7, 9 clade. Within the consensus sequences, upper-case letters indicate amino acids found in all members of a subgroup, lower-case letters indicate amino acids conserved in more than 50% of the members, pairs of lower-case amino acid in brackets show the two most abundant amino acids present for 50% each and above, x indicates that no amino acid is conserved among the sequences.

^1^Sg, MYB subgroups identified by Kranz *et al*. [18].

^2^Source of full length cDNA sequence: -a-, full length cDNA clone identified from EST of *Picea glauca *database; b- partial cDNA clone identified from EST database of *P. glauca*, extended by RACE amplifications and finally amplified as a single clone by PCR with gene specific primers, and -c-, from non degenerates primers based on *Pinus taeda MYB *sequences and used on spruce cDNA followed by RACE amplifications.

^3^Lengths are expressed in amino acid (aa) residues.

^4^The number of MYB sequences, separately from angiosperm and gymnosperms, sharing the motif among all those used in each case.

^5^The position of the motif relative to the beginning of the C-terminal domain (5' end).

Ref: references for previously reported motifs, a) Kranz *et al*. [18], b) Stracke *et al*. [31] and c) Jiang *et al*. [30] and d) new motifs.

**Table 2:** Length of spruce *MYB *coding sequences and introns with their predicted splice junctions

	Length (bp)			Intron I (phase 1)		Intron II (phase 2)	
	
	Coding sequence	Intron I	Intron II	5'Splice site	3'Splice site	5'Splice site	3'Splice site
*Pg MYB 1*	999	83	101	CCG:*GT*AAAT	TTGC*AG*:GTC	TAG:*GT*ATAT	CACC*AG*:GTG
*Pg MYB 2*	1347	501	88	CAG:*GT*ACTC	TGAC*AG*:GTC	CAG:*GT*TTGT	GTGC*AG*:GTG
*Pg MYB 3*	924	1427	none	CAG:*GT*AAAG	ATGC*AG*:GGA	none	none
*Pg MYB 4*	1005	194	267	CTG:*GT*AAGC	GTAC*AG*:GTC	CAG:*GT*TTTT	GCGC*AG*:GTG
*Pg MYB 5*	774	191	186	CAG:*GT*TGAA	TTGC*AG*:GGC	CAA:*GT*ATGT	GCGC*AG*:GTG
*Pg MYB 6*	1053	none	none	none	none	none	none
*Pg MYB 7*	1122	none	none	none	none	none	none
*Pg MYB 8*	1581	97	90	CTG:*GT*AAAG	TCGC*AG*:GCC	CAG:*GT*AATG	ACAC*AG*:GTG
*Pg MYB 9*	1251	none	none	none	none	none	none
*Pg MYB 10*	633	94	187	CAG:*GT*TTCT	ATGC*AG*:GGC	CAA:*GT*ATGT	GTGC*AG*:GTG
*Pg MYB 11*	1500	644	132	CAG:*GT*ATTT	ATGC*AG*:GAC	CAA:*GT*AAGG	TTAC*AG*:ATG
*Pg MYB 12*	1110	94	294	CAG:*GT*CACT	TTGC*AG*:GGC	CAG:*GT*GAGT	ATGT*AG*:ATG
*Pg MYB 13*	591	94	139	CAG:*GT*TTCT	ATGC*AG*:GGC	CAA:*GT*ATGT	GTGC*AG*:GTG

Coding sequences indicate the length in nucleotides from the translation start codon to the stop codon. Introns I and II represent the first and second introns, respectively. Intron phase refers to the position in a codon where the intron is inserted: after the first nucleotide (phase 1) or after the second nucleotide (phase 2) of a codon. Italic nucleotide pairs GT and AG represent the beginning and the end of the introns, respectively; and underlined nucleotides are conserved splice-site sequences.

**Table 3:** Pair-wise sequence amino acids identities of the DBD and full CDS of closest spruce and pine homologs

	DNA Binding Domains		Full coding sequences	
	
Amino acids percentage	Identity	Similarity	Identity	Similarity
PgMYB1/PtMYB1	99,1	100	87,1	91,3
PgMYB2/PtMYB2	100	100	88	91,6
PgMYB3/PtMYB3	94,6	95,4	79,2	82,3
PgMYB4/PtMYB4	96,6	97,5	84,1	88,6
PgMYB7/PtMYB7	94,8	96,5	90,4	93,6
PgMYB8/PtMYB8	98,3	100	93,1	95,5
PgMYB5/PtMYB14	88	94,8	67,3	77,4
PgMYB10/PtMYB14	87,8	95,6	64,3	73
PgMYB13/PtMYB14	82,7	92,2	60	70

Percent similarity calculations were performed using pairwise optimal alignment with Bioedit software (Clustal W, matrix blosum62).
