# Supplementary material for: Conifer R2R3-MYB transcription factors: sequence analyses and gene expression in wood-forming tissues of white spruce (Picea glauca)
Source: BMC Plant Biol. 2007 Mar 30;7:17. doi: 10.1186/1471-2229-7-17 (PMC1851958; doi:10.1186/1471-2229-7-17)
Supplement: Additional file 1 — Primers sequences of spruce MYBs used for genomic amplification and sequencing. The table provides the nucleotides sequences of the primers with respective Tm values and amplified genomics DNA length, for each spruce MYB genes. [file 1471-2229-7-17-S1.pdf]

MYB genes names	Forward primer sequences	Tm Salt Adjusted (°C)	Reverse primer sequences	Tm Salt Adjusted (°C)	Amplified gDNA length (pb)
PgMYB1	TTTAGAACGATCCCCGAATG	56	ATCTCATCCTCCTCCTCCAT	58	1336
PgMYB2	CAATGGGACGCCACTTATG	57	CTACCCTGTGAAAGACGAAGG	61	1898
PgMYB3	TTTTGAAATTGCGAACAGAATG	55	TATTTTTAGGCACCCGTTGATT	56	2430
PgMYB4	GCACGGCCATTCTATTCATT	56	CACCTGATCAAGGATCGACA	58	1503
PgMYB5	CCCTTGCAGCTAAGTTGCTT	58	TATGAATGCTTCGTGGTGGA	56	1243
PgMYB6	CTAATGGCGTCGATGAAAGG	58	GCAACCCCTGAACTCTGTGT	60	1007
PgMYB7	TGCAGAGGCTTGTTCAGAAATA	58	TTTTATTGGAAGGGCTGAGTGT	58	1220
PgMYB8	TCGATCAGAGTACGGGGATT	58	GGCTGCGAGTTTGTGTGAAT	58	1963
PgMYB9	TTCGATCCTGTTGTGAGCTG	58	CCAAGTGCCCCTTTATTTCA	56	1370
PgMYB10	CTTGGTCACGGTTTCCAATC	58	CCCATGGGATAACACACTCC	60	1022
PgMYB11	TTGCATTGTAGAGGGCCAAT	56	TCCATTCCATTCGATCTGTTC	57	2356
PgMYB12	TCAGAAGAACAGGGCACCA	57	ATAAGGGCAATGGCTGGACT	58	1507
PgMYB13	ACCTTGCACGGTCATTCTTC	58	ACACGACTTCGGGCAATTTA	56	938

Additional file 1 – Primers sequences of spruce *MYBs* used for genomic amplification and sequencing.
