# Supplementary material for: Conifer R2R3-MYB transcription factors: sequence analyses and gene expression in wood-forming tissues of white spruce (Picea glauca)
Source: BMC Plant Biol. 2007 Mar 30;7:17. doi: 10.1186/1471-2229-7-17 (PMC1851958; doi:10.1186/1471-2229-7-17)
Supplement: Additional file 2 — Phylogenetic tree of MYBs from spruce, pine and nearest sequences from other species. The figure shows the phylogenetic relationship between conifers MYBs and others MYBs on the basis of their complete amino acids sequences. Protein sequences from each clade incorporating spruce MYBs was used to search for conserved amino acid motifs. [file 1471-2229-7-17-S2.pdf]

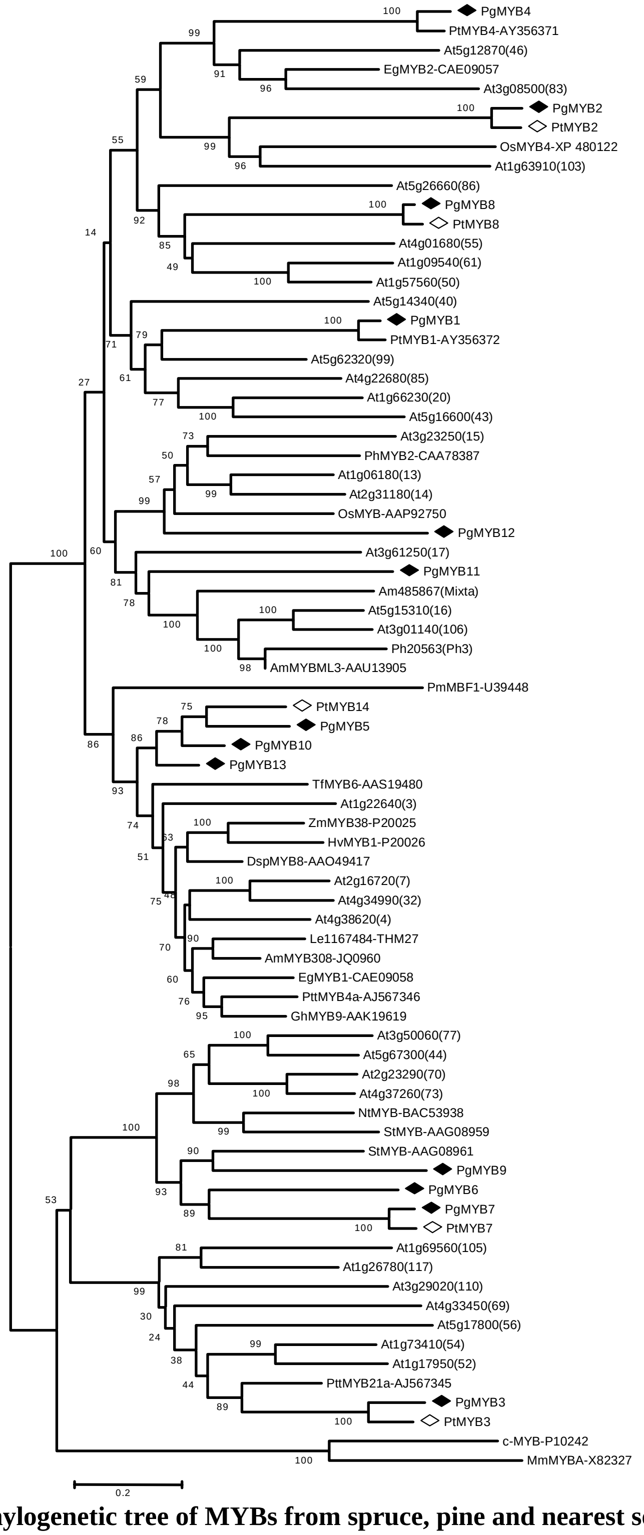

Additional file 2 – Phylogenetic tree of MYBs from spruce, pine and nearest sequences from other species

Neighbour-joining tree method (1000 Bootstraps) based on an alignment derived using the Clustal W program of the complete coding sequences of the 18 MYB proteins identified in this study (spruce and pine, with full and empty lozenge) and most similar sequences from other species. The bar indicates an evolutionary distance of 0.2%. Genomic loci are indicated for *Arabidopsis* sequences with common MYB number in brackets. For the other MYBs, the GenBank accession number is given after the MYB name except for the newly identified spruce and pine MYBs. Human c-MYB and *Mus musculus* MmMYBA were not used as out groups but as landmarks. Pg: *Picea glauca*, Pm: *Picea mariana*, At: *Arabidopsis thalianna*, Pt: *Pinus taeda*, Mm; *Mus musculus*, Human c-myb, St: *Solanum tuberosum*, Nt: *Nicotiana tabacum*, Ptt: *Populus tremuloides* x *Populus trycocarpa*, Eg: *Eucalyptus gunnii*, Os: *Oryza sativa*, Am: *Anthirinum majus*, Ph: *Petunia hybrida*, Tf: *Tradescantia fluminensis*, Hv: *Hordeum vulgare*, Gh: *Gossypium hirsutum*, Le: *Lycopersicon esculentum*, Zm: *Zea maize*, Dsp: *Dendrobium sp.*
