# Supplementary material for: Conifer R2R3-MYB transcription factors: sequence analyses and gene expression in wood-forming tissues of white spruce (Picea glauca)
Source: BMC Plant Biol. 2007 Mar 30;7:17. doi: 10.1186/1471-2229-7-17 (PMC1851958; doi:10.1186/1471-2229-7-17)
Supplement: Additional file 3 — Conifer MYB phylogeny based on partial sequences using a 31 amino acids region. The figure shows the phylogenetic relationship between several conifers MYBs on the basis of a 31 amino acid region in the third MYB domain repeat. Due to the availability of many partial sequences, this short region leads to the addition of several conifer sequences. [file 1471-2229-7-17-S3.pdf]

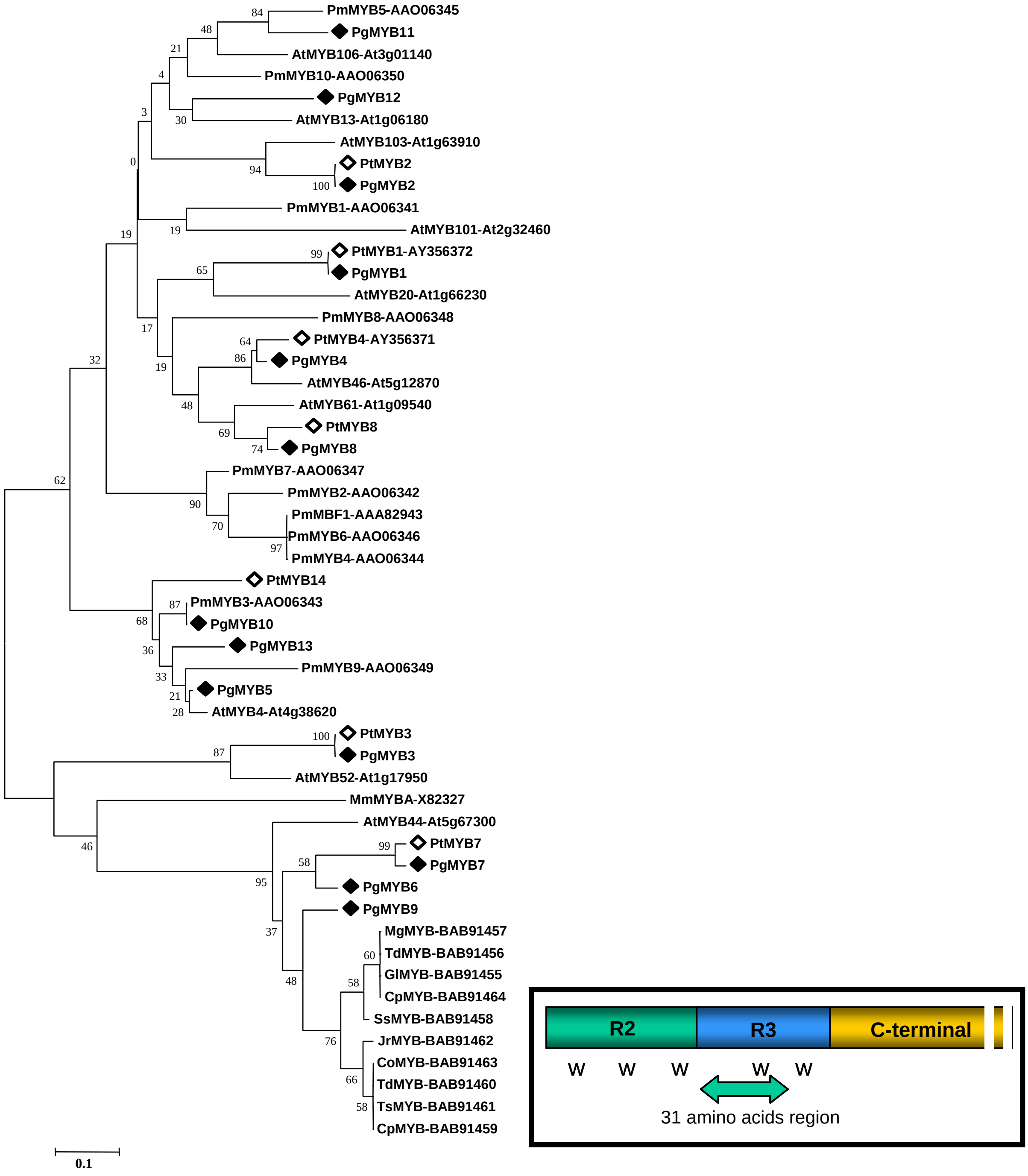

Additional file 3 – Conifer MYB phylogeny based on partial sequences using a 31 amino acids region

The 31 amino acid region in the R3 MYB domain (shown in box) is present in all the MYB used here. The phylogenetic tree was based on an alignment derived using the Clustal W program and the neighbour-joining method (1000 Bootstraps). The bar indicates an evolutionary distance of 0.1%. *Mus musculus* MmMYBA were not used as out group but as landmark. Pg: *Picea glauca*, Pm: *Picea mariana*, At: *Arabidopsis thalianna*, Pt: *Pinus taeda*, Cp: *Chamaecyparis pisifera*, Co: *Chamaecyparis obtusa*, Jr: *Juniperus rigida*, Ts: *Thuja standishii*, Td: *Thujopsis dolabrata*, Ss: *Sequoia sempervirens*, Mg: *Metasequoia glyptostroboides*, Td: *Taxodium distichum*, Gl: *Glyptostrobus lineatus*. PmMYB sequences come from Xue *et al.* [12], PtMYB1 and PtMYB4 from Patzlaff *et al.* [10, 11] and *Cupressacea* MYB sequences of the down phylogenetic clade from Kusumi *et al.* [34].
